# A Competency Assessment Tool for Liver Contrast-Enhanced Ultrasound Among Sonography Trainees: Development and Multicenter Validation Study

**DOI:** 10.2196/86730

**Published:** 2026-11-09

**Authors:** Yanping Ma, Bowen Zheng, Jie Ren, Wen He, Lei Shang, Hui Wang, Gaiqin Xue, Fang Nie, Yanli Guo, Yukun Luo, Hongxia Zhang, Xuan Wang, Xiang Jing, Ruifang Zhang, Jianjun Yuan, Rong Wu, Xiaomao Luo, Fenglin Dong, Yan Luo, Qiang Lu, Jintang Liao, Pintong Huang, Qin Chen, Jia’an Zhu, Youfeng Xu, Xiaoyan Xie

**Affiliations:** 1 Department of Medical Ultrasonics, Third Affiliated Hospital of Sun Yat-sen University, 600 Tianhe Road, Guangzhou, 510630, China, 86 13925155583; 2 Beijing Tiantan Hospital, Capital Medical University, Beijing, China; 3 Department of Health Statistics, School of Preventive Medicine, Air Force Medical University, Xi'an, China; 4 Department of Ultrasound, China-Japan Union Hospital of Jilin University, Jilin, China; 5 Shanxi Provincial Cancer Hospital, Taiyuan, China; 6 Lanzhou University Second Hospital, Lanzhou, China; 7 The First Hospital Affiliated to AMU, Chongqing, China; 8 Chinese PLA General Hospital, Beijing, China; 9 Tianjin Third Central Hospital, Tianjin, China; 10 First Affiliated Hospital of Zhengzhou University, Zhengzhou, China; 11 Henan Provincial People's Hospital, Zhengzhou, China; 12 Shanghai First People's Hospital, Shanghai, China; 13 Yunnan Cancer Hospital, Kunming, China; 14 First Affiliated Hospital of Soochow University, Soochow, China; 15 West China Hospital of Sichuan University, Sihchuan, China; 16 Xiangya Hospital Central South University, Changsha, China; 17 Second Affiliated Hospital of Zhejiang University, Hangzhou, China; 18 Sichuan Academy of Medical Sciences & Sichuan Provincial People's Hospital, Chengdu, China; 19 Peking University People's Hospital, Beijing, China; 20 The Affiliated First Hospital of Ningbo University, Ningbo, China; 21 The First Affiliated Hospital, Sun Yat-sen University, Guangzhou, China

**Keywords:** contrast-enhanced ultrasound, CEUS, competency assessment, liver, validation, training, competency-based medical education, CBME

## Abstract

**Background:**

Despite the increasing use of liver contrast-enhanced ultrasound (CEUS), no standardized, organ-specific tool exists for assessing trainees’ cognitive competency. Existing instruments lack multidimensional evaluation and external validation, which limit training quality and patient safety.

**Objective:**

This study aims to develop and validate the CEUS Competency Assessment for the Liver (CCAL), a tool to standardize the evaluation of theoretical and cognitive skills in liver CEUS.

**Methods:**

This retrospective cohort study had four stages: (1) item generation from literature and guidelines, (2) 3 Delphi rounds (12/21, 57.1% female experts; mean age 51.0, SD 7.2 y) and cognitive testing (10 trainees) for content validity (>85% agreement) and clarity, (3) internal validation involving 100 sonography trainees (n=66, 66% assistant professors; n=27, 27% associate professors; n=7, 7% professors; n=70, 70% CEUS-naive) with 1 month or more of training at 4 hospitals (2018‐2020), and (4) external validation on 579 sonography trainees (n=299, 51.6% assistant professors; n=183, 31.6% associate professors; n=97, 16.8% professors; n=439, 75.8% CEUS-naive) with 1 or more months of training at 25 multicenter sites (2018‐2020). Primary outcomes were reliability (Cronbach α and Guttman split-half) and validity (exploratory factor analysis and item discrimination), while secondary outcomes included item difficulty distribution, pass rates, and between-group differences.

**Results:**

The final CCAL comprised 74 items across 3 domains (demographics: 8, self-evaluation: 13, and objective questions: 53). Internal consistency was excellent (Cronbach α=0.916 and Guttman split-half=0.908). Factor analysis confirmed a 3-factor structure explaining 74.6% of the cumulative variance. Item analysis showed a balanced difficulty distribution (35/53, 66.0% easy; 17/53, 32.1% moderate; 1/53, 1.9% difficult items). Scores varied significantly by CEUS training duration (*P*<.001); trainees with 4 to 6 months or more than 6 months of training achieved higher self-evaluation (*F*_2,576_=34.751, partial η²=0.108, 95% CI 0.069-0.147) and objective question (*F*_2,576_=34.374, partial η²=0.107, 95% CI 0.068-0.146) scores than those with less than 3 months of training. Prior independent CEUS experience was also associated with higher scores on the self-evaluation (*t*_577_=6.736, Cohen *d*=0.561, 95% CI 0.393-0.728; *P*<.001) and objective question sections (*t*_577_=3.494, Cohen *d*=0.291, 95% CI 0.127-0.454; *P*=.001). Competency gaps were identified across specific competency domains: the pass rate ranged from 32.5% (188/579; image-guided intervention) to 81.3% (471/579; indications and contraindications).

**Conclusions:**

The CCAL is the first liver-specific CEUS competency tool integrating objective knowledge testing with structured self-evaluation. Unlike generic or simulation-based tests, it is organ-specific and validated across a large multicenter cohort, providing a multidimensional framework that identifies specific competency gaps, most notably in advanced image-guided procedures, beyond global pass/fail outcomes. The tool supports competency-based medical education through rigorous data-driven assessment, targeted curricular improvements, cross-institutional benchmarking, and informed training standards. It evaluates cognitive competence exclusively and should not replace procedural assessment in high-stakes decisions. International validation and the establishment of criterion-related and predictive validity are essential next steps.

## Introduction

### Problem

Contrast-enhanced ultrasound (CEUS) has become an indispensable diagnostic tool for liver diseases, offering real-time, radiation-free characterization of focal liver lesions and dynamic depiction of tissue microcirculation [1]. Its clinical value has been endorsed by more than 20 international guidelines from organizations such as the World Federation for Ultrasound in Medicine and Biology (WFUMB) and the European Federation of Societies for Ultrasound in Medicine and Biology (EFSUMB) [2,3]. Nevertheless, the diagnostic accuracy of CEUS is highly operator-dependent [4]. Effective application of CEUS demands high technical proficiency from operators, as the procedure is time-sensitive and susceptible to visual interference [5]. Therefore, ensuring that trainees attain standardized and objectively verifiable competencies before transitioning to independent practice is essential not only for safeguarding training quality but also, more critically, for protecting patient safety and ensuring diagnostic accuracy.

In response to this need, competency-based medical education (CBME) has emerged as a paradigm in medical training, shifting the focus from time-based curricula to demonstrable outcomes that ensure trainees acquire the knowledge, skills, and attitudes necessary for independent practice [6,7]. This approach mandates the use of standardized, validated assessment tools to objectively evaluate clinical competencies and safeguard training quality. According to the Messick unified validity framework, validity is not a property of the test itself but of the interpretations and actions based on test scores. Therefore, assessment tools must gather evidence across 5 sources—content, response process, internal structure, relations to other variables, and consequences—to support high-stakes decisions such as certification or independent practice [8,9]. However, in the field of CEUS—particularly for liver applications, which represent a core clinical scenario—there is a lack of validated assessment tools to objectively evaluate the theoretical knowledge and operational competence of trainees.

### Review of Relevant Scholarship

The importance of standardized competency assessment in medical education has been increasingly recognized. Recent systematic studies have shown that competency-based curricula require robust evaluation practices to ensure training quality, yet significant variation exists in how assessment tools are developed, implemented, and reported [10,11]. Alharbi [10] systematically reviewed evaluation practices in CBME curricula and found that, while questionnaires remain the primary assessment tool, numerous studies fail to clarify evaluation objectives, approaches, and standards, highlighting the need for more rigorous and transparent assessment methodologies. Similarly, a systematic review of undergraduate CBME assessment identified a range of constructivist techniques—including concept maps, portfolios, rubrics, and workplace-based assessments—as promising approaches, but noted that their implementation remains inconsistent and often lacks validity evidence [11].

Within the ultrasound education domain, recent years have witnessed significant efforts to establish competency frameworks. The ULTRA-Metrics study, a Delphi-derived international consensus initiative, developed a modular framework for assessing ultrasound competency across clinical applications and disciplines, identifying 3 core competency domains (experience, skills, and autonomy) and 4 skill domains (indication, acquisition, interpretation, and clinical integration) [12]. This framework highlighted that existing approaches often lack generalizability, overemphasize exam counts, and fail to assess key skills such as interpretation, thereby limiting ultrasound’s safe and effective integration into clinical practice. Similarly, Delphi consensus studies have been used to define ultrasound competencies in various specialties, including emergency medicine, anesthesiology, and radiology residency programs, reflecting a growing recognition of the need for competency-based ultrasound training [13,14]. However, these frameworks remain largely generic and have not been translated into organ-specific, validated assessment instruments.

Specifically, in the field of CEUS, several efforts have been made to develop competency assessment tools, yet substantial psychometric gaps persist. Jacobsen et al [15,16] developed a theoretical multiple-choice test for core CEUS competencies and gathered validity evidence according to the Messick framework, but the final 47-item test showed a considerable risk of false positives and false negatives when establishing a pass/fail score. The same research group also developed an immersive virtual reality simulation-based test with acceptable reliability (Cronbach’s α=0.85), yet it carried a 23.6% risk of false negatives and focused exclusively on precontrast preparations rather than on comprehensive liver CEUS competency [17]. Furthermore, a scoping search of the literature on CEUS curricula and assessment revealed very limited published work in this area, confirming the scarcity of validated assessment tools.

Beyond CEUS, the broader ultrasound education literature provides instructive parallels. A systematic review of endoscopic ultrasound (EUS) competency assessment tools identified several instruments with strong validity evidence—including TEESAT (The EUS and Endoscopic Retrograde Cholangiopancreatography Skills Assessment Tool), GAPS-EUS (Global Assessment of Performance and Skills in EUS), and EUSAT (EUS Assessment Tool)—but noted that these tools remain specialty-specific and are not directly transferable to other ultrasound applications [18]. Similarly, organ-specific ultrasound assessment tools have been developed for various applications, including lung ultrasound (LUS)–Objective Structured Assessment of Ultrasound Skills (OSAUS) and cranial ultrasound, demonstrating the feasibility of organ-specific competency assessment [19,20]. Paradoxically, although the liver is a primary indication for CEUS, accounting for the majority of global CEUS examinations, no equivalent organ-specific tool has been developed for liver CEUS competency assessment. A 2024 study on CEUS Liver Imaging Reporting and Data System (LI-RADS) classification training for radiology residents demonstrated that dedicated training improved diagnostic performance, yet no standardized assessment tool accompanied that training, underscoring a critical gap between educational intervention and rigorous outcome evaluation [21].

### Rationale and Research Questions

The convergence of the above evidence reveals a clear and urgent knowledge gap: there is currently no validated, organ-specific, and psychometrically sound competency assessment tool for liver CEUS. While generic ultrasound competency frameworks have been proposed, they lack the specificity needed for liver CEUS, which requires unique knowledge of hepatic vascular anatomy, LI-RADS classification, contrast kinetics, and image-guided interventional procedures. Furthermore, previously published CEUS tools have been limited by insufficient validity evidence (eg, undefined cutoffs) or overly narrow scope (eg, covering only precontrast preparations). This deficiency has direct clinical consequences; without robust evaluation criteria, high-stakes decisions regarding whether trainees can independently perform liver CEUS examinations become subjective, ultimately compromising patient safety and health care quality. Given the high operator dependency of CEUS and the growing clinical demand for liver examinations, developing a rigorous assessment tool is an urgent quality assurance priority.

### Hypothesis, Aims, and Objectives

To address these gaps, the present study aimed to develop and validate the first standardized competency assessment tool specifically for liver CEUS (the CEUS Competency Assessment for the Liver [CCAL]). We hypothesized that the CCAL would demonstrate the following characteristics: (1) strong content validity, as established through a Delphi expert consensus process (content evidence); (2) high internal consistency reliability and satisfactory item discriminability (internal structure evidence); and (3) robust external validity across diverse training sites (relations to other variables evidence). Accordingly, through a Delphi process involving 21 experts and a multicenter validation study comprising over 600 trainees, this study sought to provide a reliable, psychometrically sound instrument for objective competency evaluation in liver imaging, thereby filling a critical void in CBME for CEUS training.

## Methods

### Ethical Considerations

This study involved human participants and adhered to the Declaration of Helsinki. Ethical approval was obtained from the Institutional Review Board (IRB) of The Third Affiliated Hospital of Sun Yat-sen University (IRB number: [2020] 02-137-01) and was registered with the Chinese Clinical Trial Registry (ChiCTR2000035750). Written informed consent was secured from all participants prior to enrollment, and they were informed of their right to withdraw at any time without academic or other repercussions. All collected data were anonymized and deidentified before analysis, with no personal identifiers retained in the research database. Participation was voluntary and part of routine assessment procedures; therefore, no compensation was provided. The manuscript and supplementary materials contain no images or content that could identify individual participants.

### Study Procedures

#### Research Design

This study used a nonexperimental, observational, cross-sectional design with multiple-group comparisons. It was conducted from July 2020 to July 2021 and adopted a four-phase mixed methods design. It systematically followed established standards for the development of medical education assessment tools as follows: (1) item generation, (2) content refinement, (3) psychometric validation, and (4) multicenter validation [22-26]. The methodology was carefully structured to address the recognized lack of standardized competency assessment instruments for liver CEUS.

#### Phase 1: Item Generation

##### Domain Identification

The assessment domains were established based on standardized curricula from Chinese CEUS training centers and are in accordance with the 2020 WFUMB CEUS Liver Guidelines [2]. The core domains include the principles of CEUS and device optimization, diagnostic characterization of liver lesions utilizing CEUS, and management of adverse reactions.

##### Initial Item Pool Generation

Six experts in CEUS independently developed items for the self-evaluation section (SES; a 5-point Likert scale) and the objective question section (OQS; multiple-choice or single-best-answer format). The content of the OQS included indications and contraindications, scanning protocols, and imaging characteristics of liver lesions.

### Phase 2: Expert Consultation and Cognitive Testing

#### Expert Consensus Development

A 3-round modified Delphi process was conducted from September to December 2020 with 21 experts (n=12, 57.1% female experts; mean age 51.0,  SD  7.2 y; mean ultrasound experience 25.5 , SD  6.8 y; mean CEUS experience 14.8 , SD  6.0 y; Table 1). In round 1, experts rated each item on a 5-point scale and suggested additions; items achieving >85% agreement were retained, while others were revised or removed. Rounds 2 and 3 involved aggregated feedback and rerating, and the process continued until consensus was reached (Multimedia Appendix 1). Items were blindly reviewed to mitigate groupthink and ensure independence. The method integrated clinical practice needs with existing guideline recommendations [16,27].

**Table 1.** Demographic and professional characteristics of the 21 experts who participated in the 3-round Delphi consensus process for content validation of the CEUS Competency Assessment for the Liver (CCAL) conducted from September 2020 to December 2020.

Category	Values, n
Sex	
Male	9
Female	12
Administrative position	
Department director	19
Other	2
Title	
Chief physician	19
Associate chief physician	1
Associate professor	1
Academic level	
Bachelor	5
Master	3
Doctor	13
Title in the academic committee	
Chairman	9
Vice chairman	5
Others	7
Geographic distribution	
South	7
North	7
Central	4
East	3
Specialty	
Abdomen	19
Intervention	2

#### Cognitive Testing

A pilot study was conducted with 10 trainees (5 novices with <1 mo of CEUS experience, 5 experienced with >6 mo of experience) who were interviewed regarding item clarity, comprehensibility, and wording ambiguity. Their feedback was used to inform subsequent revisions.

### Phase 3: Internal Validation and Revision

#### Aim

A quantitative analysis was conducted utilizing internal validation trainees, and items of the trial version were screened to develop the final version in January 2021 [28,29].

#### Inclusion and Exclusion

Consecutive enrollment included trainees who met both of the following criteria: (1) completion of 1 or more months of CEUS training between January 2018 and December 2020 at 1 of 4 leading teaching hospitals—namely, the Chinese PLA General Hospital, Beijing Tiantan Hospital (Capital Medical University), the Second Hospital of Lanzhou University, and the Third Affiliated Hospital of Sun Yat-sen University; the training program comprised at least 30 hours per week of supervised hands-on scanning and structured didactic instruction; and (2) performance of more than 10 independent liver CEUS examinations after formal training completion. Exclusion criteria were as follows: (1) unwillingness to participate in the standardized competency assessment, and (2) prior completion of 2 or more CEUS training programs. All enrolled trainees were native Mandarin speakers and completed the assessment in Chinese. A total of 100 trainees met all eligibility criteria and were included in the study.

#### Participant Characteristics

Among 100 sonography trainees, 47% (n=47) held a bachelor’s degree, 43% (n=43) a master’s degree, and 10% (n=10) a doctorate. Professional titles included the following: 66% (n=66) assistant professors, 27% (n=27) associate professors, and 7% (n=7) full professors. Ultrasound experience was as follows: 48% (n=48) had 1‐3 years, 20% (n=20) had 4 to 5 years, and 32% (n=32) had >5 years of experience. Before training, 70% (n=70) had no independent CEUS experience. CEUS training duration was as follows: 58% (n=58) trained for 1 to 3 months, 24% (n=24) for 4 to 6 months, and 18% (n=18) for more than 6 months. The mean number of CEUS examinations performed per trainee was 81.71 (SD 112.96; Multimedia Appendix 2).

#### Sampling Procedures

Participants were recruited consecutively from the 4 predetermined teaching hospitals. No random or stratified sampling was used; every eligible trainee was invited to participate. The participation rate was 100% (100/100) because the assessment was part of the routine training completion process. Self-selection at the individual level did not occur; institutional self-selection did not apply, as the 4 hospitals were preselected collaborators. No compensation was provided.

#### Sample Size, Power, and Precision

For internal validation, the sample size was determined pragmatically based on the number of eligible trainees available at the 4 sites during the study period (January 2018-December 2020). A minimum of 100 participants was considered adequate for initial item screening and exploratory factor analysis, following established recommendations for scale development (subject-to-item ratio ≥5:1). The achieved sample of 100 trainees exceeded this threshold for the OQS items (ratio ≈1.9:1, which is lower than the recommended 5:1; however, this was acceptable for preliminary validation and was compensated by the larger external validation sample).

#### Measures and Covariates

Primary measures were the SES (5-point Likert scale) and the OQS (multiple-choice or image-based). Covariates included age, sex, academic degree, professional title, years of ultrasound experience, prior independent CEUS experience (yes or no), CEUS training duration (mo), and the number of CEUS examinations performed during training. All covariates were collected using an 8-item demographic questionnaire during the assessment.

#### Data Collection and Quality of Measurements

The trial version was hosted online. Trainees accessed the assessment via a direct link and completed all items independently, with webcam monitoring and page-switching prohibited to prevent cheating. All participants were given a uniform time limit of 60 minutes. The website automatically recorded and stored the results and calculated the scores. The dataset contained no missing values; all enrolled participants fully completed the assessment, and no imputation methods were applied.

### Phase 4: External Validation

#### Aim

To further expand the scope of validation, a multicenter external validation study was conducted in April 2021 [24].

#### Inclusion and Exclusion

Consecutive enrollment included trainees who met both of the following criteria: (1) completion of 1 month or more of CEUS training between January 2018 and December 2020 at 25 leading teaching centers distributed across multiple regions of China; the training program comprised at least 30 hours per week of supervised hands-on scanning and structured didactic instruction; and (2) performance of more than 10 independent liver CEUS examinations after formal training completion. The same exclusion criteria were applied. All enrolled trainees were native Mandarin speakers and completed the assessment in Chinese.

#### Participant Characteristics

Among the 579 sonography fellows, the majority held a bachelor’s degree (n=336, 58.0%), were CEUS-naive before training (n=439, 75.8%), and received 1 to 3 months of CEUS training (n=337, 58.2%; Table 2). The mean number of CEUS examinations performed per trainee during training was 64.72 (SD 107.81).

**Table 2.** Demographic and clinical characteristics of trainees (N=579) in the external validation phase (phase 4) of the contrast-enhanced ultrasound (CEUS) competency assessment for the liver (CCAL) development study across 25 multicenter sites in China for liver CEUS competency assessment conducted in April 2021.

Characteristics	Values, n (%)
Sex	
Male	125 (21.6)
Female	454 (78.4)
Academic level	
Bachelor	336 (58.0)
Master	185 (31.9)
Doctor	58 (10.0)
Title	
Assistant professor	299 (51.6)
Associate professor	183 (31.6)
Professor	97 (16.8)
Years engaged in ultrasound	
1‐3	204 (35.2)
4-5	89 (15.4)
>5	286 (49.4)
Had independent CEUS examination experience before training	
Yes	140 (24.2)
No	439 (75.8)
Duration of CEUS training (mo)	
1‐3	337 (58.2)
4‐6	116 (20.0)
>6	126 (21.8)

#### Sampling Procedures

Participants were recruited consecutively from 25 multicenter sites that agreed to participate (out of 45 invited; response rate n=25, 55.6%). At the trainee level, all eligible individuals were approached, and the participation rate was 100% (579/579). No individual self-selection occurred because the assessment was part of routine training completion. At the institutional level, self-selection was possible (centers chose to participate or decline). No compensation was provided.

#### Sample Size, Power, and Precision

Sample size was determined a priori using a 10:1 subject-to-item ratio for the final 53 OQS items, requiring at least 530 participants. The external validation cohort included 579 participants, exceeding the required sample size.

#### Measures and Covariates

The same measures (SES and OQS) and covariates (demographic characteristics, prior experience, training duration, and number of CEUS exams) were used as in phase 3. All covariates were collected via an 8-item demographic questionnaire during the assessment.

#### Data Collection and Quality of Measurements

Same as the internal validation part mentioned above.

### Analytic Strategy

#### The Primary Outcomes

The primary outcomes included the following: (1) internal consistency reliability, with Cronbach α and the Guttman split-half coefficient ≥0.8 considered acceptable; (2) construct validity, assessed via exploratory factor analysis (principal axis factoring with varimax rotation). The Kaiser-Meyer-Olkin (KMO) measure of sampling adequacy was >0.8, and Bartlett test of sphericity was significant (*P*<.001), indicating that the data were suitable for factor analysis. The extracted factor structure was examined for alignment with the theoretically hypothesized structure; and (3) the index of discrimination (D), evaluated by the correlation between each item score and the total OQS score, where items were retained only if the discrimination index D was ≥0.4 [30,31].

#### The Secondary Outcomes

The secondary outcomes included the following:

1. The distribution of item difficulty (ie, the proportion of easy, moderate, and difficult items). The difficulty index (H) was used to describe the difficulty of the OQS, where H of 0.3 or less indicated easy questions, 0.3<H<0.7 indicated moderately difficult questions, and H of 0.7 or more indicated difficult questions [32-34]. H was calculated as 1–P, where P is the proportion of correct responses.
2. Pass rates for each knowledge domain.
3. Between-group differences in overall OQS scores across trainee subgroups (eg, stratified by duration of CEUS training or prior clinical experience).

#### Item Analysis

Items exhibiting low difficulty (H≤0.3) or poor discrimination (D≤0.2) were removed after the first phase (n=13 items), which resulted in the final item set.

#### Statistical Analysis

All statistical analyses were conducted using SPSS Statistics (version 26.0; IBM). Continuous variables are presented as the mean (SD), and categorical variables are presented as the frequency and percentage. The group comparisons used *t* tests, ANOVA, or nonparametric tests (Mann-Whitney *U* test and Kruskal-Wallis test) on the basis of data type and distribution. Associations were assessed with chi-square tests and Pearson correlations (*P*<.05, 2-tailed).

## Results

### Participant Flow

In phase 1, 6 experts generated items independently without formal enrollment or attrition tracking. In phase 2, all 21 invited experts and 10 recruited trainees completed the Delphi process and cognitive interviews, respectively, with no attrition. For phase 3 internal validation, 111 trainees met the eligibility criteria; 11 declined participation, and the remaining 100 were enrolled and completed the assessment in full, with no missing data or further withdrawals. In phase 4 external validation across 25 hospitals, 646 trainees were assessed for eligibility; 67 declined participation, and 579 were enrolled and completed the assessment with no missing data or withdrawals (Figure 1).

**Figure 1.**
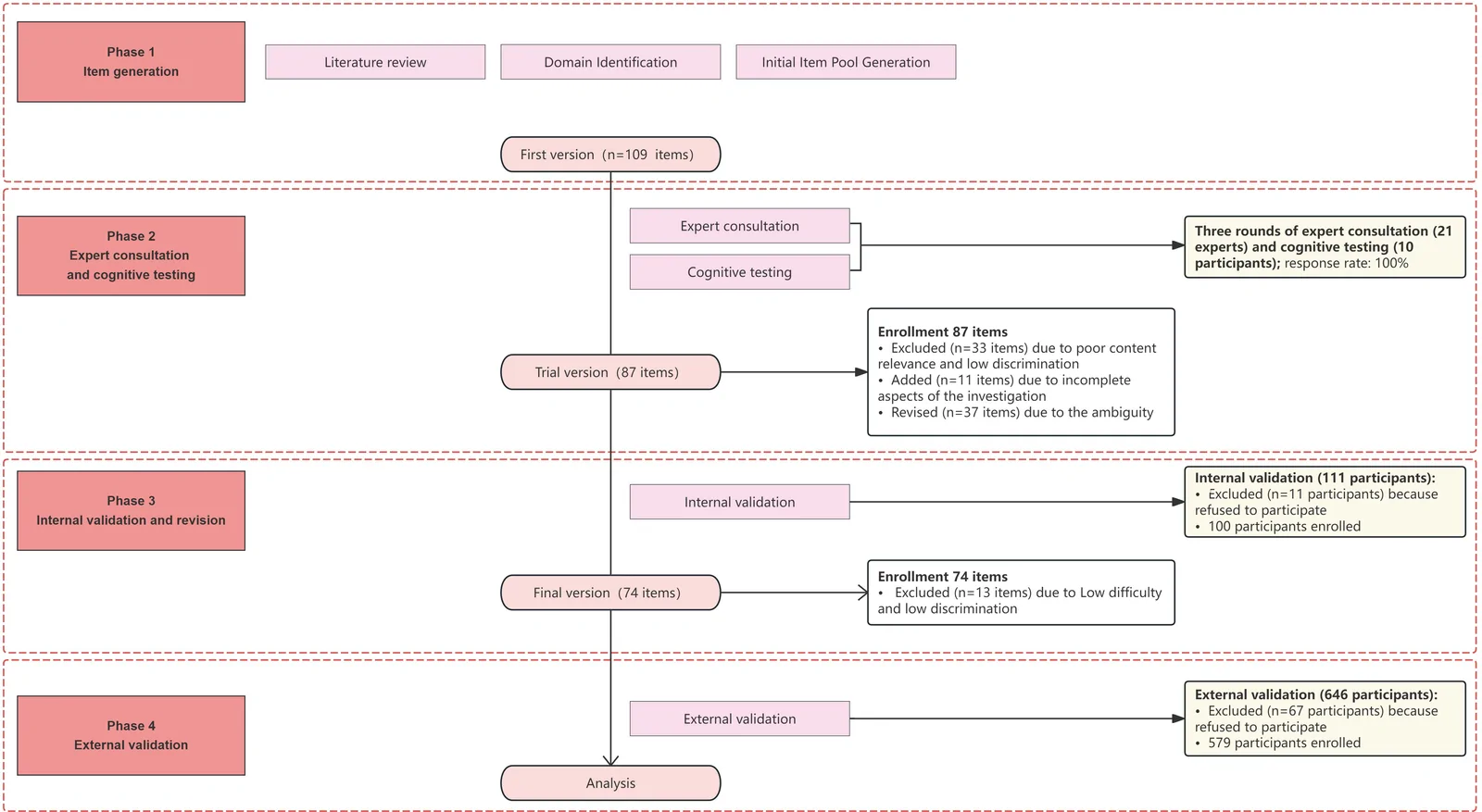
Retrospective study design and participant flow diagram of the 4-phase development and validation process of the contrast-enhanced ultrasound (CEUS) Competency Assessment for the Liver (CCAL) for trainees in liver CEUS, conducted across 25 multicenter sites in China from July 2020 to July 2021.

### Recruitment

Recruitment was conducted in 4 phases. Phase 1 (item generation) took place in July 2020. Phase 2 (expert consultation and cognitive testing) ran from September to December 2020. Phase 3 (internal validation) was conducted in January 2021 at 4 preselected teaching hospitals. Phase 4 (external validation) occurred in April 2021. The study used a cross-sectional design with no repeated measures or follow-up.

### Statistics and Data Analysis

#### Item Selection Process

To address the critical gap in the objective assessment of liver CEUS competency, this study developed the CCAL. An initial pool of 109 items (demographic: 8; SES: 24; OQS: 77) was reduced to a final version of 74 items (demographic: 8; SES: 13; OQS: 53) following 3 rounds of optimization (expert consultation, cognitive interviews, and internal validation). The final items span 3 clinically relevant domains: technical proficiency (eg, scanning protocols and device operation), diagnostic decision-making (eg, lesion characterization and interpretation), and clinical judgment (eg, indication selection and complication management).

#### Psychometric Validation

The final scale was then systematically evaluated across multiple dimensions using an external validation cohort of 579 trainees.

##### Reliability

Reliability analysis demonstrated excellent internal consistency: Cronbach α=0.916 and Guttman split-half coefficient=0.908, indicating strong intercorrelations among items and consistent measurement of the construct.

##### Construct Validity

Construct validity was supported by a KMO of 0.921 and a significant Bartlett test (*P*<.001), confirming suitability for factor analysis and alignment with clinical competency requirements.

The scree test extracted 3 common factors, accounting for 74.6% of the cumulative variance, with all item factor loadings above 0.67. Factor 1 represents technical proficiency and encompasses device optimization and contraindication recognition, which reflect procedural execution capability. Factors 2 and 3 jointly capture diagnostic efficacy, specifically the improvement in diagnostic accuracy, which reflects interpretive competence and clinical decision-making skills (Table 3). This empirically derived factor structure strongly corresponds to the requirements of clinical competency.

**Table 3.** Factor loadings and dimension attribution of the self-evaluation section (SES) of the CEUS Competency Assessment for the Liver (CCAL) during the external validation phase (phase 4) among 579 trainees from 25 centers in China for the liver contrast-enhanced ultrasound (CEUS) competency assessment conducted in April 2021.

Items	Factor loading	Dimension attribution
Do you think it is necessary to perform CEUS examination in patients with suspected intrahepatic malignant lesions for the first time?	0.786	3
Do you think it is necessary to perform CEUS examination in patients with suspicious intrahepatic benign lesions for the first time?	0.855	3
During CEUS, I can find lesions that are not visible on 2D ultrasound.	0.672	2
I believe I have mastered the principles of CEUS technology.	0.900	1
I believe I have mastered the adjustment of the instruments during CEUS.	0.910	1
I believe I have mastered the indications and contraindications for CEUS.	0.863	1
I believe I can handle the adverse effects of CEUS with confidence.	0.854	1
I can use CEUS to qualitatively diagnose the vast majority of target lesions in general.	0.734	1
I believe CEUS has significantly improved the accuracy of my diagnosis.	0.857	2
I believe that the CEUS images and report descriptions of mine are normative.	0.832	1
I believe CEUS can improve the positive rate of tissue biopsy of mine.	0.837	2
How well do you think you have mastered the CEUS examination?	0.817	1
In order to accurately diagnose target lesions with comprehensive information, CEUS physicians need to be proficient in reading CT^a^ and MRI^b^ images.	0.754	2

a CT: computed tomography.

b MRI: magnetic resonance imaging.

##### Discriminant Validity

Discriminant validity was examined to determine whether the scale distinguishes trainees by competence level. Scores differed significantly by experience and professional rank. Specifically, trainees with 4 or more months of CEUS training scored higher on both self-evaluation (*F*_2,576_=34.751; partial η²=0.108, 95% CI 0.069-0.147) and objective questions (*F*_2,576_=34.374; partial η²=0.107, 95% CI 0.068-0.146) than those with less than 3 months (*P*<.001). Additionally, those with prior independent CEUS experience also scored higher on both the self-evaluation (*t*_577_=6.736; *P<*.001; Cohen *d*=0.561, 95% CI 0.393-0.728) and objective question sections (*t*_577_=3.494; *P*=.001; Cohen *d*=0.291, 95% CI 0.127-0.454). These findings confirm that the scale effectively differentiates trainees by ability level (Figure 2**;** Multimedia Appendices 3 and 4).

**Figure 2.**
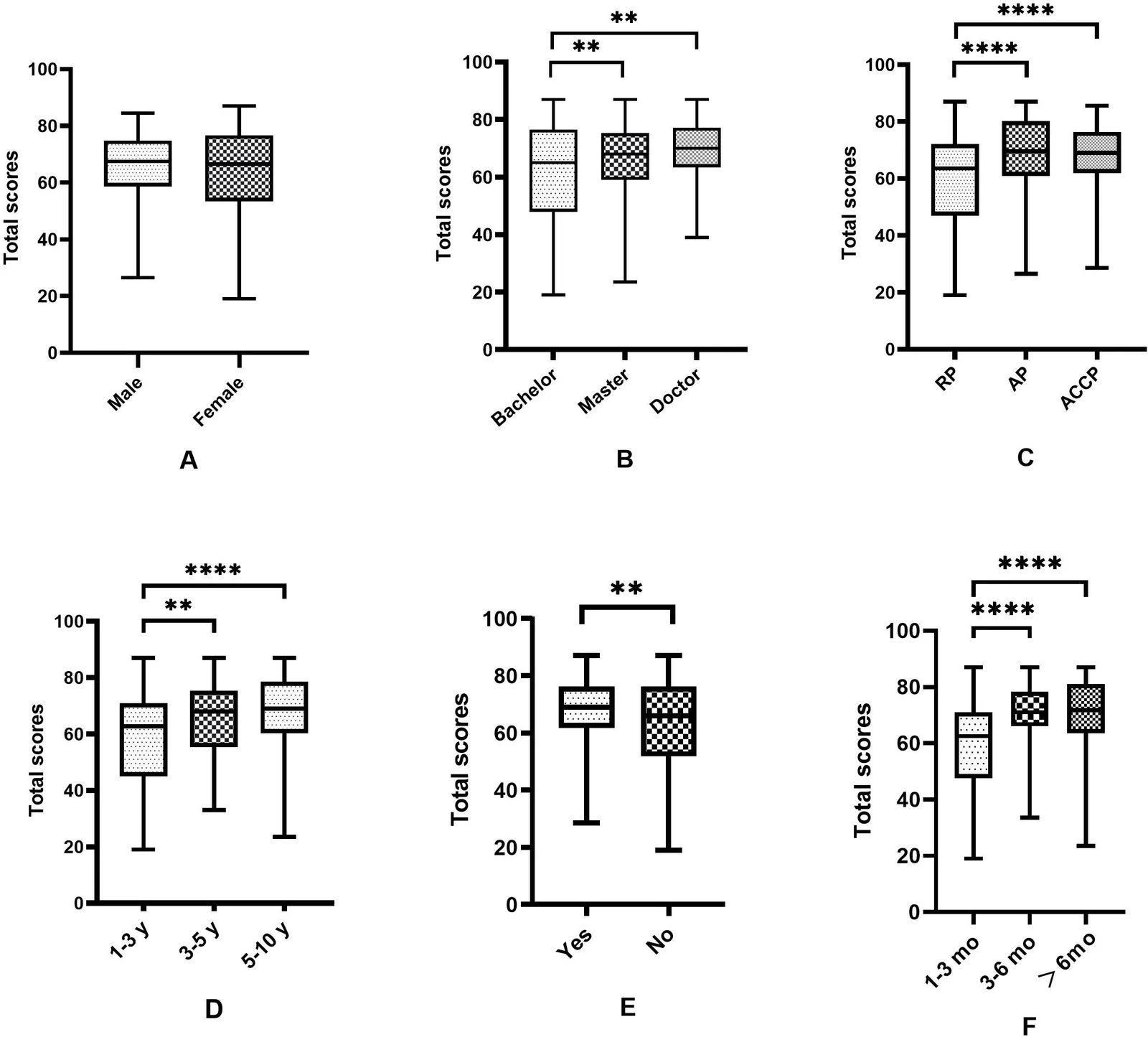
Box plots of total objective question section scores by trainee characteristics among 579 trainees in the external validation phase (phase 4) of the multicenter study of the Contrast-Enhanced Ultrasound Competency Assessment for the Liver (CCAL) for competency assessment in liver contrast-enhanced ultrasound, conducted in April 2021. (A) Total scores grouped by sex (male: fine black dot fill; female: coarse black‑and‑white checkerboard fill). (B) Total scores grouped by academic level (bachelor: fine black dot fill; master: coarse black‑and‑white checkerboard fill; doctor: fine grey‑and‑white checkerboard fill). (C) Total scores grouped by title (RP: resident physician, fine black dot fill; AP: assistant professor, coarse black‑and‑white checkerboard fill; ACCP: associate chief physician and chief physician, fine grey‑and‑white checkerboard fill). (D) Total scores grouped by years engaged in ultrasound (1-3 y: fine black dot fill; 3-5 y: coarse black‑and‑white checkerboard fill; 5-10 y: fine grey‑and‑white checkerboard fill). (E) Total scores grouped by prior independent CEUS experience (yes: fine black dot fill; no: coarse black‑and‑white checkerboard fill). (F) Total scores grouped by duration of CEUS training (1-3 mo: fine black dot fill; 3-6 mo: coarse black‑and‑white checkerboard fill; >6 mo: fine grey‑and‑white checkerboard fill). The ** denotes *P*<.01 and **** denotes *P*<.0001.

Spearman correlation analysis revealed a significant positive correlation between trainees’ objective question scores and self-evaluation scores (rs=0.551; *P*<.001).

### Difficulty

The OQS demonstrated excellent discrimination, with a discrimination index of 0.429, exceeding the established threshold for good discrimination (D≥0.4). Moreover, the OQS items were appropriately distributed across difficulty levels, covering a broad range of trainee ability: 66.0% (35/53, 95% CI 0.526‐0.773) of items were categorized as simple questions (covering basic knowledge points), 32.1% (17/53, 95% CI 0.211‐0.455) as medium-level questions (pertaining to clinical applications), and 1.9% (1/53, 95% CI 0.003‐0.099) as challenging questions (involving advanced skills).

### Identification of Weaknesses in Competency

Quantitative analysis of module-specific pass rates (Table 4) identified critical competency gaps: the pass rate for “indications and contraindications” was 81.3% (471/579, 95% CI 0.780‐0.843), indicating solid command of fundamental knowledge, whereas the pass rate for “imaging features for procedure guidance” was only 32.5% (188/579, 95% CI 0.288‐0.364), highlighting insufficient training in CEUS application for clinical procedures.

**Table 4.** Comparison of scores across different content domains (indications, scanning protocols, imaging features, and procedural guidance) of the objective question section (OQS) in the external validation phase (phase 4) among 579 trainees from 25 Chinese centers for liver contrast-enhanced ultrasound (CEUS) competency in April 2021.

Category	Indications and contraindications		Scanning procedures and general operating steps	Imaging features of space-occupying lesions in the liver		Imaging features used to guide puncture, tumor ablation, or other procedures.	
	Scores, mean (SD)	Pass rate, % (95% CI)	Scores, mean (SD)	Scores, mean (SD)	Pass rate, % (95% CI)	Scores, mean (SD)	Pass rate, % (95% CI)
Sex							
Male	12.73 (2.16)	81.6 (0.739‐0.874)	13.58 (3.00)	35.38 (9.02)	79.2 (0.713‐0.854)	4.56 (2.40)	38.4 (0.303‐0.472)
Female	12.47 (2.57)	81.3 (0.774‐0.846)	12.69 (3.28)^a^	34.58 (10.27)	78.6 (0.746‐0.822)	4.94 (2.26)	30.8% (0.268‐0.352)
Academic level							
Bachelor	12.31 (2.70)	85.7^b^ (0.816‐0.891)	12.29 (3.41)	33.18 (11.02)	78.9 (0.742‐0.829)	4.77 (2.29)	32.4 (0.277‐0.376)
Master	12.82 (2.18)	75.7 (0.690‐0.813)	13.54 (2.81)^c^	36.42 (8.37)^c^	81.1 (0.748‐0.861)	4.92 (2.37)	30.3 (0.241‐0.372)
Doctor	12.85 (1.92)	74.1 (0.616‐0.837)	14.18 (2.73)^c^	38.54 (6.17)^c^	70.7 (0.580‐0.808)	5.19 (2.04)	39.7 (0.281‐0.525)
Title							
Assistant professor	12.25 (2.61)	73.6^b^ (0.683‐0.783)	12.24 (3.40)	32.18 (10.68)	74.2^b^ (0.690‐0.789)	4.52 (2.28)	29.8 (0.249‐0.352)
Associate professor	12.94 (2.40)^d^	92.9 (0.882‐0.958)	13.64 (2.94)^d^	37.54 (8.53)^d^	85.8 (0.799‐0.901)	5.30 (2.40)^d^	33.9 (0.274‐0.410)
Professor	12.61 (2.17)	83.5 (0.749‐0.896)	13.45 (2.87)^d^	37.46 (8.32)^d^	79.4 (0.703‐0.862)	5.08 (1.94)	38.1 (0.291‐0.484)
Years engaged in ultrasound							
1‐3	11.88 (2.51)	72.5^b^ (0.661‐0.782)	11.84 (3.37)	31.32 (11.23)	74.5^b^ (0.681‐0.800)	4.40 (2.29)	32.4 (0.263‐0.390)
4‐5	12.74 (2.54)^e^	79.8 (0.703‐0.868)	13.34 (3.20)^e^	35.12 (9.14)^e^	74.2 (0.642‐0.821)	5.00 (2.32)	29.2 (0.208‐0.394)
>5	12.93 (2.37)^e^	88.1 (0.838‐0.914)	13.48 (2.97)^e^	37.09 (8.59)^e^	83.2 (0.785‐0.871)	5.15 (2.24)^e^	33.6 (0.284‐0.392)
Independent CEUS experience before training?							
Yes	12.79 (2.20)	83.6 (0.766‐0.888)	13.58 (2.93)	37.14 (7.93)	76.4 (0.688‐0.827)	5.02 (1.96)	32.9 (0.256‐0.410)
No	12.44 (2.57)	80.6 (0.767‐0.844)	12.66 (3.30)^f^	34.00 (10.49)^f^	79.5 (0.755‐0.830)	4.80 (2.39)	32.3 (0.281‐0.369)
Duration of CEUS training (mo)							
1‐3	11.93 (2.49)	77.4^b^ (0.727‐0.816)	12.00 (3.20)	32.29 (10.63)	75.7^b^ (0.708‐0.799)	4.46 (2.24)	32.1 (0.273‐0.372)
4‐6	13.09 (1.78)^g^	93.1 (0.870‐0.965)	14.13 (2.36)^g^	38.84 (6.35)^g^	86.2 (0.788‐0.913)	5.59 (2.10)^g^	40.5 (0.320‐0.496)
>6	13.60 (2.59)^g^	81.0 (0.732‐0.869)	14.08 (3.30)^g^	37.60 (9.10)^g^	80.2 (0.724‐0.862)	5.25 (2.38)^g^	26.2 (0.193‐0.345)
Total	12.53 (2.50)	81.3 (0.780‐0.8431)	12.88 (3.24)	34.76 (10.01)	78.7 (0.752‐0.819)	4.86 (2.29)	32.5 (0.288‐0.364)

a *P*<.05 compared with male participants.

b *P*<.05.

c *P*<.05 compared with participants with a bachelor’s degree.

d *P*<.05 compared with participants who were assistant professors.

e *P*<.05 compared with participants who engaged in ultrasound for 1-3 years.

f *P*<.05 compared with participants without prior independent CEUS examination experience.

g *P*<.05 compared with participants who received CEUS training for 1-3 months.

## Discussion

### Principal Results

This study developed and validated the first organ-specific competency assessment tool for liver CEUS. Following a 3-round Delphi process and cognitive interviews, the CCAL was tested in internal and external multicenter cohorts. It demonstrated strong content validity, high internal consistency reliability, and robust construct and discriminant validity. Subdomain analysis revealed a pronounced competency gap in image-guided interventions, confirming the tool’s ability to identify specific educational needs beyond global pass or fail outcomes.

### Interpretations, Implications, and Comparison With Prior Work

The CCAL advances the field of CEUS competency assessment through several innovations that directly address the limitations of existing tools. Jacobsen et al [15,16] laid important groundwork by developing a core CEUS syllabus and operationalizing it into a 47-item multiple-choice test; however, their instrument was explicitly “not recommended for certification purposes” due to the risk of false positives and negatives, and its utility was limited to entry-level screening. The same research group later developed an immersive virtual reality simulation-based test focusing on precontrast preparations, yet it carried a 23.6% risk of false negatives and did not address comprehensive liver CEUS competency [17]. More broadly, other sonographic assessments, such as the OSAUS or generic radiology in-training examinations, assess general scanning skills or factual knowledge but lack the organ-specific, multidimensional construct needed to capture the unique demands of liver CEUS, including contrast-agent safety, dynamic enhancement patterns, and image-guided interventional decision-making [12,35,36]. These tools, while pioneering, were not organ-specific and lacked a self-assessment component, leaving an unmet need for a multidimensional, liver-dedicated assessment.

The CCAL addresses this gap through 5 defining strengths. With respect to content validity and theoretical foundation, item generation followed the 2020 WFUMB liver CEUS guidelines and was refined through a 3-round Delphi process involving 21 experts, which was followed by cognitive interviews, establishing the CCAL as the first liver-specific, expert-consensus-driven CEUS competency tool [2,37]. This expert-consensus approach stands in marked contrast to assessments developed in isolation by a single institution or those lacking systematic, evidence-informed item vetting. The high internal consistency and a 3-factor structure consistent with the theoretical blueprint confirm that the CCAL measures a coherent yet multidimensional construct—encompassing theoretical knowledge, image interpretation, and clinical judgment—rather than a single global ability. In comparison, prior CEUS tests either reported no reliability data or were confined to narrow procedural tasks [15-17]. The demonstration of known-group discriminant validity, with scores distinguishing trainees by experience and professional title, supports the tool’s use for benchmarking progress along a developmental continuum, a critical requirement for competency-based education [38,39]. Methodologically, the CCAL used a 4-phase mixed methods design and is the first tool to integrate objective knowledge testing with structured self-evaluation within a single instrument, enabling multidimensional assessment of theoretical knowledge, self-awareness, and clinical decision-making rather than a unidimensional pass or fail judgment [40,41]. Finally, the online platform with remote proctoring and automated scoring ensures that the CCAL can be deployed at low cost across diverse clinical settings, lowering the barriers to standardized assessment in resource-limited environments [42].

Beyond global pass-or-fail decisions, the CCAL offers granular diagnostic capability through subdomain analysis. Pass rates were high in fundamental areas such as indications and contraindications but dropped substantially for advanced competencies, like image-guided navigation for biopsy and tumor ablation. This finding aligns with published training challenges in interventional ultrasound, where the cognitive load of integrating real-time imaging with needle manipulation is high and structured curricula are often lacking [43]. The ability to pinpoint such a specific competency gap enables program directors to target educational interventions, such as simulation-based training modules or dedicated rotations, rather than applying blanket remediation [44,45]. This function aligns with the principles of assessment for learning, whereby diagnostic information directly informs curricular improvement [46].

The incorporation of structured self-evaluation enhances the comprehensiveness and validity of the assessment. A statistically significant positive correlation between objective assessment and self-evaluation scores indicates that trainees demonstrate reasonable metacognitive awareness of their clinical competence. However, the presence of notable outliers—individuals who consistently overestimate or underestimate their performance by a clinically meaningful margin—raises important patient safety concerns and warrants targeted remediation [46,47]. Overestimation in image-guided procedures could lead to premature independent practice and an increased risk of complications [48]. Conversely, underestimation may unnecessarily erode confidence and limit procedural engagement [49]. By flagging such discrepancies, the CCAL enables supervisors to provide targeted feedback that fosters accurate self-assessment—a skill increasingly recognized as essential for lifelong learning and safe unsupervised practice [50,51]. This dual-modality reporting thus moves beyond traditional knowledge tests that offer no window into trainees’ metacognitive awareness [52].

Collectively, these characteristics position the CCAL as a practical tool for implementing CBME in CEUS training. Standardized, multisource data on cognitive competence can inform entrustment decisions, document training progression for accreditation, and facilitate cross-institutional quality assurance, thereby addressing the persistent reliance on subjective, uncalibrated assessments that currently dominate the field. While the CCAL explicitly does not assess hands-on procedural skills and should not be used in isolation for high-stakes credentialing, within its intended scope, it provides a transparent, evidence-based foundation for evaluating and advancing the cognitive component of liver CEUS competence.

### Limitations

This study had the following limitations. First, the validity evidence chain remains incomplete because of the absence of predictive validity verification—specifically, the correlation between test scores and real-time clinical decisions during procedures (eg, video-based assessments or supervisor ratings [53,54]). Second, easy items predominate (66% of the objective section), which may limit discrimination among advanced practitioners, although this was intentional given the target population (novices to competent operators) [55]. Third, standardization frameworks require further development as follows: norm-referenced benchmarks (eg, 60% as the passing threshold for first-year residents) remain unestablished, and institutional variations necessitate multilevel modeling to delineate confounding factors (eg, trainer expertise and equipment availability) [56]. Fourth, the CCAL was validated only in Chinese trainees across 25 Chinese hospitals; generalizability to non-Chinese populations (different curricula, certification, patient populations, and cultural self-assessment tendencies) remains unproven, requiring international validation [57,58]. Fifth, the relationship between self-perceived competency in the SES and objectively measured clinical performance needs further study, as we did not include direct observation (eg, live scanning or video-recorded exams)—the gold standard for criterion validity [41,59]. Future research should examine concurrent validity against supervisor ratings or objective structured assessment of technical skills (OSATS) during actual patient encounters [60]. Collectively, these advantages—content validity grounded in expert consensus, high reliability, robust construct validity, multicenter validation, a multidimensional construct, and fine-grained diagnostic capacity—constitute clear innovations over prior tools, while the acknowledged limitations delineate a focused agenda for future research.

### Conclusions

The CCAL is the first organ-specific competency assessment tool for liver CEUS, innovatively combining objective knowledge testing with structured self-evaluation. Unlike generic or simulation-based CEUS tests, it is liver-specific and validated across a large multicenter cohort. It brings a standardized, refined framework that identifies specific competency gaps. Practically, the tool supports CBME by enabling data-driven assessment, enhancing rigor in CEUS examinations, guiding targeted curricular improvements, permitting cross-institutional benchmarking, and supplying evidence for specialty societies to update training standards, though further validation is required before high-stakes use. It must be emphasized that the CCAL evaluates theoretical competence exclusively and should not replace procedural assessment for certification decisions. Within its intended scope, the CCAL advances liver CEUS training toward greater standardization and evidence-based feedback. International validation and the establishment of criterion-related and predictive validity are essential next steps.

## Supplementary material

10.2196/86730 Multimedia Appendix 1 Results of the 3-round Delphi expert consultation for content validation of the Contrast-Enhanced Ultrasound Competency Assessment for the Liver.

10.2196/86730 Multimedia Appendix 2 Demographic and clinical characteristics of trainees in the internal validation phase of the Contrast-Enhanced Ultrasound Competency Assessment for the Liver development study.

10.2196/86730 Multimedia Appendix 3 Univariate and multivariate analyses of factors associated with self-evaluation section (SES) scores in the external validation cohort.

10.2196/86730 Multimedia Appendix 4 Factors associated with objective question section (OQS) scores in the external validation cohort.

## Abbreviations

CBME: competency-based medical education
CCAL: Contrast-Enhanced Ultrasound Competency Assessment for the Liver
CEUS: contrast-enhanced ultrasound
EFSUMB: European Federation of Societies for Ultrasound in Medicine and Biology
EUS: endoscopic ultrasound
EUSAT: Endoscopic Ultrasound Assessment Tool
GAPS-EUS: Global Assessment of Performance and Skills in Endoscopic Ultrasound
IRB: Institutional Review Board
KMO: Kaiser-Meyer-Olkin
LI-RADS: Liver Imaging Reporting and Data System
LUS: lung ultrasound
OQS: objective question section
OSATS: objective structured assessment of technical skills
OSAUS: objective structured assessment of ultrasound skills
SES: self-evaluation section
TEESAT: The Endoscopic Ultrasound and Endoscopic Retrograde Cholangiopancreatography Skills Assessment Tool
WFUMB: World Federation for Ultrasound in Medicine and Biology
