# Supplementary material for: A Competency Assessment Tool for Liver Contrast-Enhanced Ultrasound Among Sonography Trainees: Development and Multicenter Validation Study
Source: JMIR Med Educ. 2026 Nov 9;12:e86730. doi: 10.2196/86730 (PMC13747318; doi:10.2196/86730)
Supplement: Supplementary file 1 [file mededu-v12-e86730-s001.docx]

**Supplementary Table 1. Results of the three‑round Delphi expert consultation for content validation of the CCAL from September to December 2020** **.**

| Phase | Judgment Coefficient (Ca) | Familiarity Coefficient (Cs) | Authority Coefficient (Cr) | Variation Coefficient  (CV) | Kendall's Coefficient of Concordance  (Kendall's W) |
| --- | --- | --- | --- | --- | --- |
| 1 | 0.89 | 0.76 | 0.78 | 0.18 | 0.134 |
| 2 | 0.86 | 0.94 | 0.90 | 0.16 | 0.250 |
