# Supplementary material for: A Competency Assessment Tool for Liver Contrast-Enhanced Ultrasound Among Sonography Trainees: Development and Multicenter Validation Study
Source: JMIR Med Educ. 2026 Nov 9;12:e86730. doi: 10.2196/86730 (PMC13747318; doi:10.2196/86730)
Supplement: Supplementary file 2 [file mededu-v12-e86730-s002.docx]

**Supplementary Table 2. Demographic and clinical characteristics of trainees (N=100) in the internal validation phase (Phase 3) of the CCAL development study across four centers in China for liver CEUS competency assessment in January 2021.**

| Characteristics | n | Percentage（%） |
| --- | --- | --- |
| Sex |  |  |
| Male/ Female | 25/75 | 25/75 |
| Academic level |  |  |
| Bachelor/ Master / Doctor | 47/43/10 | 47/43/10 |
| Title |  |  |
| Assistant professor/ Associate professor/ Professor | 66/27/7 | 66/27/7 |
| Years engaged in Ultrasound |  |  |
| 1-3 Years/4-5 Years/＞5 Years | 48/20/32 | 48/20/32 |
| Had independent CEUS examination experience before training | |  |
| YES/NO | 30/70 | 30/70 |
| Duration of CEUS Training |  |  |
| 1-3months/4-6months/＞6months | 58/24/18 | 58/24/18 |
