# Supplementary material for: A Competency Assessment Tool for Liver Contrast-Enhanced Ultrasound Among Sonography Trainees: Development and Multicenter Validation Study
Source: JMIR Med Educ. 2026 Nov 9;12:e86730. doi: 10.2196/86730 (PMC13747318; doi:10.2196/86730)
Supplement: Supplementary file 3 [file mededu-v12-e86730-s003.docx]

**Supplementary Table 3. Univariate and multivariate analysis of factors influencing the mean self‑evaluation section (SES) scores among trainees (N= 579) in the multicenter external validation cohort (Phase 4) for liver CEUS competency in April 2021.**

| **Category** | **Factor 1** | **Factor 2** | **Factor 3** | **Total scores** | **OR（95% CI)** | ***p*** |
| --- | --- | --- | --- | --- | --- | --- |
| **Sex** |  |  |  |  |  |  |
| Male | 20.06±5.45 | 14.01±2.13 | 5.55±1.53 | 39.62±7.58 |  |  |
| Female | 19.22±6.31 | 13.69±2.51 | 5.37±0.85 | 38.28±8.55 |  |  |
| **Academic level** |  |  |  |  |  |  |
| Bachelor | 18.79±6.46 | 13.51±2.56 | 5.33±1.46 | 37.63±8.70 |  |  |
| Master | 19.82±5.74 | 14.02±2.31 | 5.53±1.51 | 39.37±7.75 |  |  |
| Doctor | 21.59±4.85^a^ | 14.33±1.77 | 5.53±1.57 | 41.45±7.39^a^ |  |  |
| **Title** |  |  |  |  |  |  |
| Assistant professor | 17.39±6.38 | 13.30±2.61 | 5.15±1.52 | 35.84±8.50 |  |  |
| Associate professor | 21.47±5.49^b^ | 14.42±2.06^b^ | 5.72±1.34^b^ | 41.62±7.46^b^ |  |  |
| Professor | 21.69±4.21^b^ | 13.89±2.22 | 5.65±1.49^b^ | 41.23±6.55^b^ |  |  |
| **Years engaged in Ultrasound** |  |  |  |  |  |  |
| 1-3 Years | 16.96±6.84 | 13.03±2.79 | 5.16±1.57 | 35.15±8.97 | 1.00 |  |
| 4-5 Years | 18.74±6.37^ce^ | 13.70±2.42 | 5.20±1.46^e^ | 37.64±8.83^ce^ | 1.54（0.89-2.65） | 0.122 |
| ＞5Years | 21.35±4.74^cd^ | 14.29±1.99^c^ | 5.66±1.38^cd^ | 41.30±6.66^cd^ | 3.41（2.28-5.10） | ＜0.01 |
| **Had independent CEUS examination**  **experience before training?** |  |  |  |  |  |  |
| Yes | 21.98±4.16 | 14.13±2.07 | 5.90±1.50 | 42.00±6.30 |  |  |
| No | 18.58±6.44^f^ | 13.64±2.53^f^ | 5.28±1.44^f^ | 37.47±8.64^f^ |  |  |
| **Duration of CEUS Training** |  |  |  |  |  |  |
| 1-3 months | 17.77±6.59 | 13.26±2.61 | 5.22±1.53 | 36.25±8.78 | 1.00 |  |
| 4-6 months | 21.93±4.38^g^ | 14.47±1.65^g^ | 5.53±1.30^g^ | 41.94±5.89^g^ | 2.51（1.59-3.96） | ＜0.01 |
| ＞6months | 21.44±4.76^g^ | 14.43±2.20^g^ | 5.81±1.41^g^ | 41.67±7.01^g^ | 3.33（2.13-5.22） | ＜0.01 |

a: *p*<0.05 compared with participants with a bachelor's degree;

b: *p*<0.05 compared with participants who were Assistant professors;

c: *p*<0.05 compared with participants who engaged in ultrasound for 1-3 years;

d: *p*<0.05 compared with participants who engaged in ultrasound for 3-5 years;

e: *p*<0.05 compared with participants who engaged in ultrasound for more than 5 years;

f: *p*<0.05 compared with participants with independent CEUS examination experience before training;

g: *p*<0.05 compared with participants who received CEUS training for 1-3 months;
