# Supplementary material for: A Competency Assessment Tool for Liver Contrast-Enhanced Ultrasound Among Sonography Trainees: Development and Multicenter Validation Study
Source: JMIR Med Educ. 2026 Nov 9;12:e86730. doi: 10.2196/86730 (PMC13747318; doi:10.2196/86730)
Supplement: Supplementary file 4 [file mededu-v12-e86730-s004.docx]

**Supplementary Table 4. Univariate and multivariate analysis of factors influencing the mean objective question section (OQS) scores among trainees (N= 579) in the multicenter external validation cohort (Phase 4) for liver CEUS competency in April 2021.**

| **Category** | **Theoretical Investigation** | **Practical**  **Investigation** | **Case Analysis** | **Total scores** | **OR（95% CI)** | ***p*** |
| --- | --- | --- | --- | --- | --- | --- |
| **Sex** |  |  |  |  |  |  |
| Male | 15.12±2.70 | 36.26±8.71 | 13.58±4.10 | 64.96±13.73 |  |  |
| Female | 14.33±3.13^a^ | 35.89±9.90 | 13.22±4.57 | 63.44±15.81 |  |  |
| **Academic level** |  |  |  |  |  |  |
| Bachelor | 14.02±3.34 | 34.60±10.62 | 12.76±4.70 | 61.38±16.83 |  |  |
| Master | 15.12±2.54^b^ | 37.36±8.06^b^ | 13.88±4.21^b^ | 66.36±13.06 ^b^ |  |  |
| Doctor | 15.30±2.35^b^ | 39.45±6.51^b^ | 14.57±3.32 ^b^ | 69.31±9.92 ^b^ |  |  |
| **Title** |  |  |  |  |  |  |
| Assistant professor | 14.03±3.26 | 33.45±10.18 | 12.54±4.58 | 60.02±16.23 |  |  |
| Associate professor | 15.07±2.80^c^ | 38.72±8.42^c^ | 14.29±4.37^c^ | 68.08±13.85^c^ |  |  |
| Professor | 14.89±2.67^c^ | 38.53±7.99^c^ | 13.79±3.88^c^ | 67.21±12.35^c^ |  |  |
| **Years engaged in Ultrasound** | |  |  |  |  |  |
| 1-3 Years | 13.69±3.30 | 32.75±10.61 | 11.92±4.47 | 58.36±16.58 | 1.00 |  |
| 4-5 Years | 14.87±2.79^d^ | 36.32±9.15^d^ | 13.70±4.49^d^ | 64.89±14.64^d^ | 1.95（1.12-3.41） | 0.019 |
| ＞5Years | 14.97±2.85^d^ | 38.16±8.41^d^ | 14.16±4.23^d^ | 67.28±13.58^d^ | 2.01（1.15-3.51） | 0.014 |
| **Had independent CEUS examination experience before training?** | | | |  |  |  |
| Yes | 14.85±2.67 | 38.21±7.64 | 14.11±3.97 | 67.17±12.12 |  |  |
| No | 14.39±3.17 | 35.25±10.12^e^ | 13.04±4.59^e^ | 62.69±16.16^e^ |  |  |
| **Duration of CEUS Training** | |  |  |  |  |  |
| 1-3 months | 13.75±3.16 | 33.60±10.20 | 12.18±4.18 | 59.53±15.70 | 1.00 |  |
| 4-6 months | 15.44±2.12^f^ | 40.01±6.59^f^ | 14.78±3.55^f^ | 70.23±10.18^f^ | 2.97（1.85-4.76） | ＜0.01 |
| ＞6months | 15.64±2.95^f^ | 38.58±8.61^f^ | 14.95±5.06^f^ | 69.17±14.83^f^ | 3.01（1.91-4.75） | ＜0.01 |

a: *p*<0.05 compared with male;

b: *p*<0.05 compared with participants with a bachelor's degree;

c: *p*<0.05 compared with participants who were Assistant professors;

d: *p*<0.05 compared with participants who engaged in ultrasound for 1-3 years;

e: *p*<0.05 compared with participants with independent CEUS examination experience before;

f: *p*<0.05 compared with participants who received CEUS training for 1-3 months;
